# Latest assessment on COVID-19 from the European Centre for Disease Prevention and Control (ECDC)

**DOI:** 10.2807/1560-7917.ES.2020.25.8.2002271

**Published:** 2020-02-27

**Authors:** Eurosurveillance editorial team

**Affiliations:** 1 European Centre for Disease Prevention and Control (ECDC), Stockholm, Sweden

The European Centre for Disease Prevention and Control (ECDC) provides regularly updated information on coronavirus disease-2019 (COVID-19) relevant to Europe through a dedicated webpage. ECDC also publishes regular risk assessments. Because of the rapidly changing situation in Europe, we provide a summary from the one published today in the Box below.

Box **. ECDC daily risk assessment on COVID-19, 27 February 2020**

- The risk associated with COVID-19 infection for people in the EU/EEA and UK is currently considered to be low to moderate.
- The risk of the occurrence of similar clusters, similar to the ones in Italy, associated with COVID-19 in other countries in the EU/EEA and the UK is currently considered to be moderate to high.
- The risk for people from the EU/EEA and the UK travelling/resident in areas with presumed community transmission is currently high.
- The risk for healthcare systems capacity in the EU/EEA and the UK during the peak of the influenza season is low to moderate.

COVID-19: coronavirus disease 2019; EU/EEA: European Union/European Economic Area; UK: United Kingdom.

We would also like to remind the readers of *Eurosurveillance *about our dedicated ‘Coronavirus disease 2019 (COVID-19) collection’ that we update regularly with new articles. 

